# Resistome and Mobilome Profiling of Raw Cow and Buffalo Milk from the Brazilian Amazon via Shotgun Metagenomics

**DOI:** 10.3390/antibiotics15050454

**Published:** 2026-04-30

**Authors:** Paulo Alex Machado Carneiro, Lenita Ramires dos Santos, Rodrigo Jardim, Christian Barnadd Danniell Gomes e Silva, Flábio Ribeiro de Araújo, Alberto Martín Rivera Dávila

**Affiliations:** 1 Amazonas State Federal Institute of Science and Technology (IFAM), Manaus 69075-351, AM, Brazil; paulo.carneiro@ifam.edu.br; 2 Embrapa Beef Cattle, Campo Grande 79106-550, MS, Brazil; lenita.santos@embrapa.br (L.R.d.S.); flabio.araujo@embrapa.br (F.R.d.A.); 3 Computational and Systems Biology Lab, Oswaldo Cruz Institute (Fiocruz), Rio de Janeiro 21040-900, RJ, Brazil; jardim@ioc.fiocruz.br; 4 Federal Superintendency of Agriculture of Amazonas, Ministry of Agriculture, Livestock and Supply (SFA-MAPA), Manaus 69057-010, AM, Brazil; christian.barnadd@agro.gov.br

**Keywords:** antimicrobial resistance, raw milk, shotgun metagenomics, water buffalo, Amazon, one health, mobile genetic elements

## Abstract

**Background/Objectives:** Antimicrobial resistance (AMR) is a global health threat, with raw milk serving as a potential reservoir for antimicrobial resistance genes (ARGs) and mobile genetic elements (MGEs). This study characterized the resistome and mobilome of raw milk from cows (*Bos taurus*) and water buffalo (*Bubalus bubalis*) in the Brazilian Amazon, a region where unpasteurized dairy consumption is culturally ingrained. **Methods:** Using shotgun metagenomic sequencing, we analyzed 32 pooled milk samples from extensive and semi-intensive farms in the Manaus Metropolitan Region. **Results:** Sequencing yielded over 3.1 million contigs. While cow milk showed a higher prevalence of positive samples (80%), buffalo milk exhibited a significantly higher abundance and diversity of ARG-associated contigs (301 contigs vs. 85 in cows). Clinically relevant genes were identified, including *AbaQ*, *ArnT*, and *KpnF*, alongside complex multi-AMR cassettes co-occurring with plasmids and widespread viral sequences (dominated by *Caudoviricetes*). Integrons were ubiquitous in cattle and highly prevalent in buffalo samples. **Conclusions:** These findings indicate that raw milk in the Amazon harbors a rich reservoir of resistance determinants and MGEs, likely driven by farm-level antibiotic usage. This underscores a critical food safety risk and highlights the need for One Health-based surveillance in the region.

## 1. Introduction

Antimicrobial resistance (AMR) constitutes a critical global public health challenge, with antimicrobial resistance genes (ARGs) increasingly recognized as the primary mechanisms enabling bacterial survival against therapeutic treatments [1]. Food-producing animals act as significant reservoirs of ARGs, which can be transmitted to humans through the food chain. In this context, livestock-derived products, particularly raw milk, serve as key vehicles for the introduction of ARGs into the human microbiota. This transmission is frequently facilitated by mobile genetic elements (MGEs), including plasmids, transposons, and integrons, which mediate horizontal gene transfer (HGT) among bacterial species [1,2]. Recent reviews within the One Health framework emphasize the interconnected dissemination of AMR across human, animal, and environmental sectors [1,3]. Raw milk is a complex biological matrix hosting diverse microbial communities, ranging from commensals to opportunistic pathogens and bacteria harboring ARGs. Consequently, the consumption of unpasteurized milk and dairy products has been linked to the transmission of ARGs to humans, prompting growing interest in characterizing the milk “resistome”, the full complement of resistance genes present. Studies have documented ARGs in raw milk destined for human consumption [4], and a recent systematic review confirmed the worldwide persistence and diversity of ARGs in milk at the farm level [5].

The role of MGEs in propagating ARGs is particularly concerning. Elements such as plasmids, integrons, transposons, and bacteriophages enable rapid HGT across different hosts and environments [6,7]. In ruminants, MGEs are abundant and functionally diverse within gastrointestinal microbiomes [8], raising concerns regarding their presence in milk and their contribution to AMR transmission, especially in regions where raw milk consumption is prevalent.

In Brazil, the sale of raw milk is regulated by Decree-Law No. 923 (1969), which restricts the direct sale of unpasteurized milk to areas where pasteurized alternatives are unavailable [9]. Despite this legislation, enforcement remains inconsistent, particularly in rural and remote regions. Due to the vast continental distances of the Amazon and the strong cultural traditions, enforcing the 1969 law is very difficult, rendering the informal sale of raw milk the main source of income for many small producers. Consequently, raw milk is widely consumed across Brazil, often in traditional forms, posing potential public health risks. While historical studies on Brazilian milk microbiology have focused on specific pathogens such as *Mycobacterium bovis* [10,11,12], *Staphylococcus aureus* [13,14,15], *Streptococcus agalactiae* [16], *Listeria monocytogenes* [17], *Brucella* spp. [18], and *E. coli* [19] comprehensive data on the overall microbial community structure, ARG diversity, and the prevalence of MGEs remain scarce, particularly in the Amazon region.

In the state of Amazonas, raw milk and its derivatives, such as clotted milk (coalhada) and artisanal cheeses (queijo coalho), are deeply embedded in local dietary customs. In this context, dairy production is not limited to cattle; water buffalo (*Bubalus bubalis*) farming is highly adapted to the Amazonian flooded plains and represents a cornerstone of the regional dairy sector [12]. Given the widespread informal consumption of raw milk in Amazonas and the paucity of AMR data in this region, we hypothesized that raw cow and buffalo milk harbor diverse microbial communities containing ARGs associated with MGEs. The objectives of this study were to (i) characterize the taxonomic profile of raw milk using shotgun metagenomic sequencing (SMS), (ii) determine the prevalence and diversity of ARGs, and (iii) in vestigate their association with MGEs.

## 2. Results

### 2.1. Sequencing Output and Assembly

**Figure 1:**
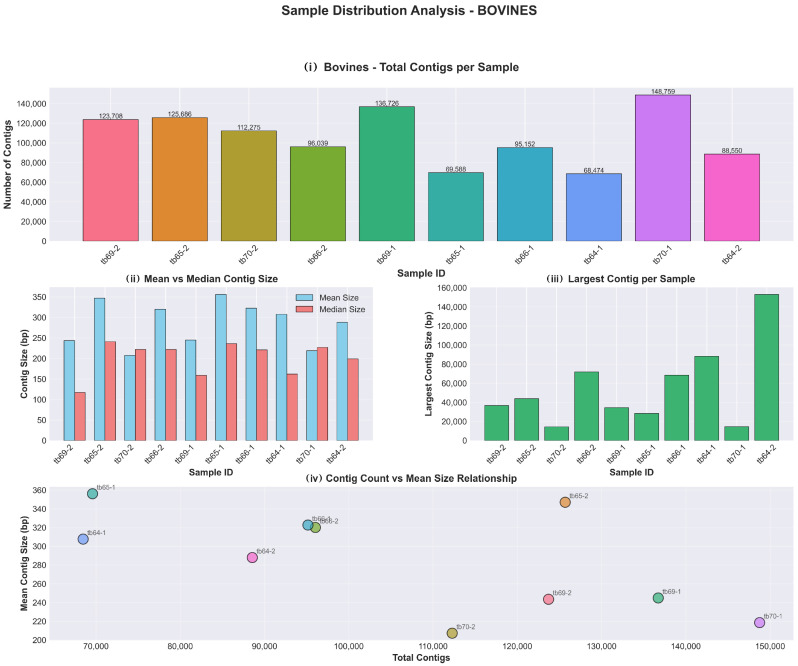
Bovines sample distribution: (**i**) total contigs per sample, (**ii**) mean and median contig size, (**iii**) largest contig per sample and (**iv**) contig count vs. mean size relationship.

**Figure 2:**
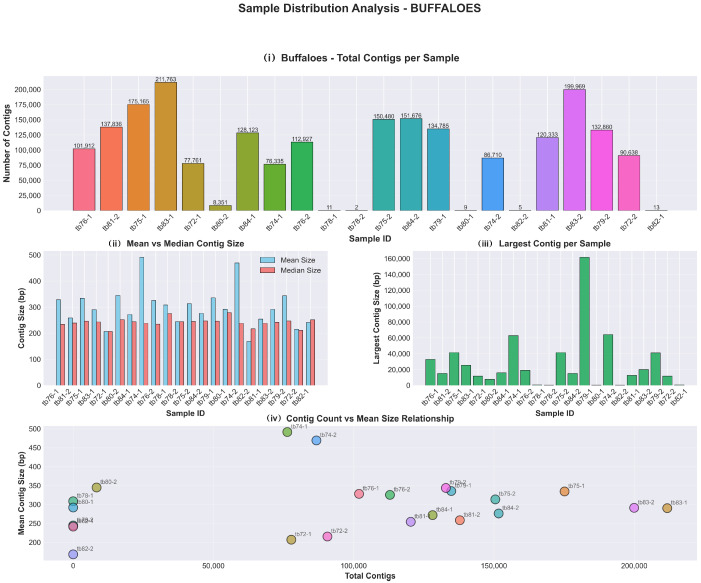
Buffaloes sample distribution: (**i**) total contigs per sample, (**ii**) mean and median contig size, (**iii**) largest contig per sample and (**iv**) contig count vs. mean size relationship.

**Figure 3:**
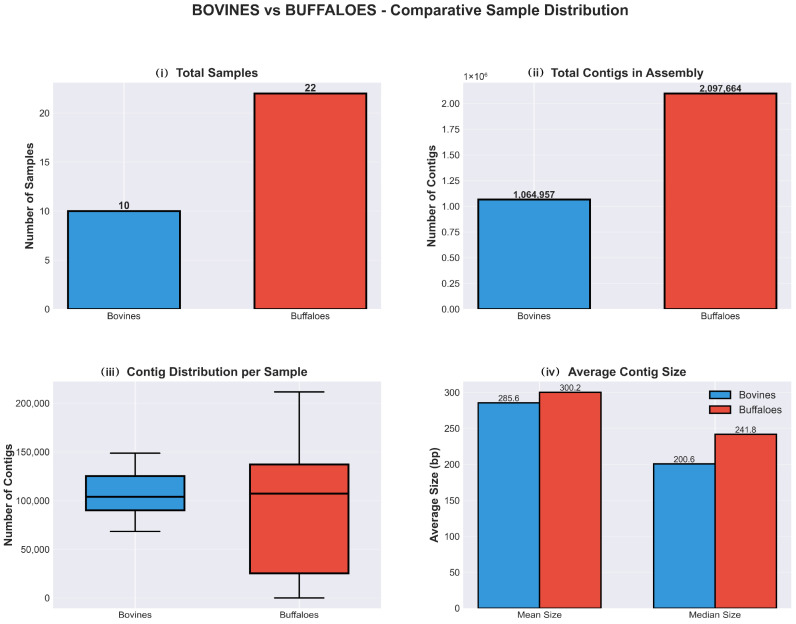
Bovines-Buffaloes comparative sample distribution: (**i**) total samples, (**ii**) total contigs in assembly, (**iii**) contigs distribution per sample and (**iv**) average contig size.

A total of 3,162,621 high-quality contigs were obtained from the raw milk samples. Sequencing yield varied significantly by host species. Cattle samples (*n* = 10) generated 1,064,957 contigs with a mean contig size ranging from 207.3 bp to 356.1 bp. The results are presented in Figure 1. Buffalo samples (*n* = 22) yielded 2,097,664 contigs with mean sizes between 168.2 bp and 491.3 bp. The results are presented in Figure 2. Annotation coverage was approximately 0.8% for cattle and 1.0% for buffalo samples. Although both groups exhibited very low coverage of annotated contigs with results (~0.8–1.0%), buffaloes yielded 21,560 contigs with results compared to 8779 in bovines, a 2.5-fold difference despite a two-fold difference in total contigs. The results are presented in Figure 3 and Supplementary Data containing a synthesis of all analysis results per group has been deposited at Harvard Dataverse (https://doi.org/10.7910/DVN/2GV0TY).

### 2.2. Antimicrobial Resistance Genes (ARGs)

**Figure 4:**
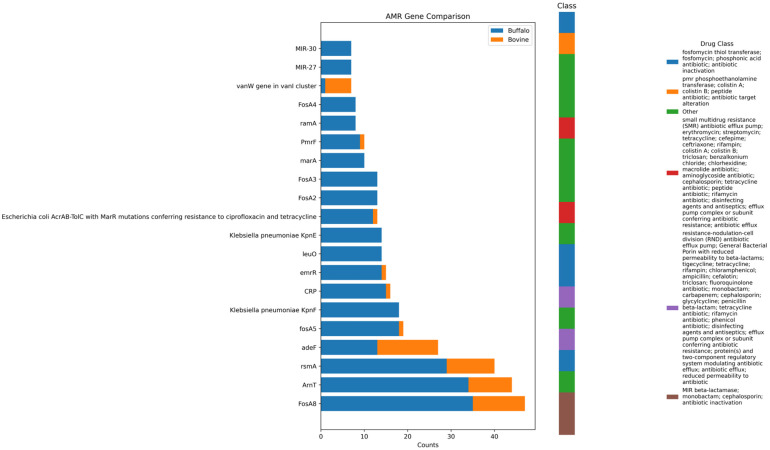
Bovine and Buffalos antimicrobial resistance gene comparison and distribution with emphasis on drug class.

Antimicrobial resistance genes were widely distributed across the dataset. Although cattle samples showed a higher frequency of positive samples, buffalo samples exhibited a greater total load and diversity of resistance markers. The results are presented in Figure 4 and Supplementary Data containing all the AMR analysis results has been deposited at Harvard Dataverse (https://doi.org/10.7910/DVN/2GV0TY). In cattle, ARGs were detected in 80% (8/10) of samples, corresponding to 85 contigs. Multi-AMR genes were identified in 50% of these samples, with the most frequent profile involving the *vanW* gene cluster (*vanB*, *vanG*, *vanI*). Conversely, buffalo samples showed ARGs in 72.7% (16/22) of samples, totaling 301 contigs. Multi-AMR genes were also found in 50% of buffalo samples, but with greater complexity; combinations involving *Klebsiella pneumoniae* efflux pumps (*KpnE*, *KpnF*) and *Escherichia coli* genes (*AcrAB*-*TolC*, *marA*) were prevalent.

### 2.3. The Mobilome: Integrons, Viruses, and Plasmids

The analysis of mobile genetic elements revealed widespread potential for horizontal gene transfer. Integrons were ubiquitous in cattle samples (100% prevalence, 26 contigs) and highly prevalent in buffalo samples (68.2%, 86 contigs). Regarding viral communities, bacteriophages dominated the milk virome in both species, with the class *Caudoviricetes* accounting for over 95% of sequences. Buffalo samples exhibited nearly threefold higher viral richness (4895 contigs) compared to cattle (1750 contigs). Plasmid sequences were detected by both geNomad and PlasmidHunter, with the highest abundance in buffalo samples (peaking in sample 74-2). Notably, no contigs classified as plasmids by the prediction tools directly carried ARGs. However, given the highly fragmented nature of the short-read assembly, this lack of direct physical linkage is likely a bioinformatics artifact rather than a true biological absence, as ARGs were frequently assembled into isolated short contigs separated from their corresponding plasmid backbones.

### 2.4. Co-Occurrence and Rare Events

Genetic co-occurrence of ARGs, plasmids, integrons, and viruses was observed across the dataset. In cattle, one sequence (sample 64-1) simultaneously harbored AMR genes, plasmid elements, and viral signatures. Buffalo samples showed even greater complexity, with 65 co-occurring sequences detected. Rare events included the identification of *ArnT* and *AbaQ* in cattle, while buffalo samples contained rare determinants such as *KpnF*, *KpnE*, *leuO*, and *MdtQ*. Rare viral classes, including *Megaviricetes*, *Herviviricetes*, and *Alsuviricetes*, were detected in single instances.

### 2.5. Multi-AMR Gene Combinations

**Figure 5:**
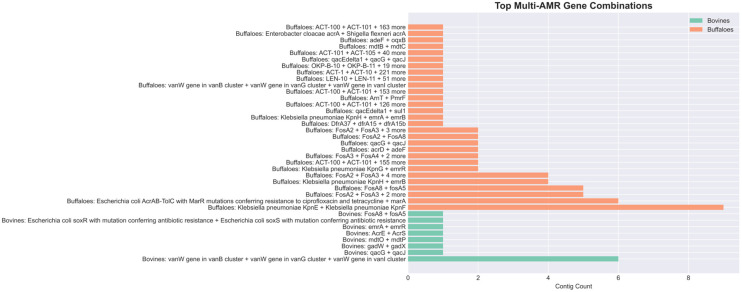
Top multi-antimicrobial resistance genes combinations distributed among bovines and buffaloes, showing clearly a more complex scenario in buffaloes.

Sequences harboring multiple antimicrobial resistance (AMR) genes were detected in both cattle and buffalo milk. In cattle, a total of 13 contigs with multiple AMR genes were identified across 5 samples. Combinations included the *vanW* cluster, *qacG + qacJ*, and *AcrE* + *AcrS*. In buffaloes, 63 contigs with multiple AMR genes were identified, with prominent combinations including *KpnE* + *KpnF*, *FosA2* + *FosA3*, and *KpnH* + *emrB*. The results are presented in Figure 5.

### 2.6. No-Results Analysis

Despite the detection of diverse elements, a significant proportion of sequences lacked database matches. In cattle, 1,056,178 of 1,064,957 sequences (99.18%) distributed among the 10 samples lacked matches, while in buffaloes, 2,076,104 of 2,097,664 sequences (98.97%) distributed among 22 samples produced no hits. All 10 (100%) bovine and 16 (72.7%) buffalo samples analyzed had at least one contig with results.

## 3. Discussion

Cattle and buffalo farming are strategic to the economy of Amazonas State [20]. However, the warm, humid climate increases microbial contamination risks. While pasteurization is critical for food safety, the consumption of raw milk cheese remains culturally significant [21,22]. Although quantitative data on antibiotic consumption could not be obtained due to the informal nature of these farming operations, our observational findings strongly suggest that current farm-level practices—ranging from extensive to semi-intensive systems with limited veterinary oversight and empirical over-the-counter antibiotic use, likely drive the selective pressure for ARGs. The incomplete or unmonitored treatment of conditions such as mastitis and omphalitis facilitates the persistence of diverse ARGs and MGEs in the milk microbiome. This dynamic is consistent with observations in other developing regions, where informal farming practices and unregulated access to veterinary drugs have been widely identified by other researchers as primary drivers of complex, unpredicted resistomes in raw dairy products [23].

This study provides a comprehensive characterization of the resistome and mobilome of raw milk from the Amazon. A key finding is the distinction between host species: while cattle milk showed high sample prevalence (80.0%), buffalo milk consistently exhibited a richer diversity and higher total abundance of ARG-associated contigs (totaling 301 vs. 85 in cattle). This aligns with reports suggesting that buffalo milk supports a richer microbial diversity and metabolic potential [24], which may favor the persistence of accessory genes, including AMR determinants. Furthermore, our hypothesis that host species acts as a major selective filter is corroborated by comparative metagenomic studies across different dairy species, which consistently demonstrate that variations in milk composition and host immunology distinctly shape the resident microbiome and its associated resistome [25]. Sequencing yielded 3,162,621 contigs across 32 samples, but more than 99.04% of contigs produced no hits to AMR genes, viruses, plasmids, or integrons, suggesting a low prevalence of these elements in the analyzed microbial communities relative to the total biomass.

Furthermore, the inherent challenges of short-read shotgun metagenomics in complex dairy matrices resulted in highly fragmented assemblies, with short mean contig sizes (168–491 bp) and over 99% of the sequences lacking database matches (“microbial dark matter”). Consequently, the resistome and mobilome characterized in this study likely represent a conservative baseline of the known risk profile rather than the absolute biological reality. This fragmentation significantly hindered the ability to perform robust genomic binning to recover Metagenome-Assembled Genomes (MAGs). Because standard binning algorithms require contigs substantially larger than our assembly’s mean size (168–491 bp) to accurately cluster sequences based on tetranucleotide frequencies, we could not definitively link specific ARGs to their complete bacterial hosts. However, the detection of lineage-specific resistance determinants, such as *KpnE*/*KpnF* (*K. pneumoniae*) and *AcrAB*-*TolC* (*E. coli*), provides strong circumstantial evidence of opportunistic pathogens contributing to the raw milk resistome. To overcome these limitations, future investigations should incorporate long-read sequencing technologies (e.g., Oxford Nanopore or PacBio). Achieving highly contiguous assemblies will be essential to accurately bridge ARGs with host genomes and to map the complete architecture of multi-AMR plasmids and integrons within the milk microbiome.

Notably, our results contrast with recent findings in organized dairy production systems. A 2025 study on cattle milk and feces from organized farms reported distinct resistome profiles driven largely by standardized management and hygiene protocols [26]. In that study, the resistome was heavily influenced by fecal contamination but showed specific clusters associated with therapeutic interventions common in intensive farming. In contrast, our Amazonian dataset—derived from extensive and semi-intensive farms with irregular veterinary support—reveals a more chaotic resistome profile. The presence of broad-spectrum efflux pumps and opportunistic pathogen-associated genes in our samples likely reflects environmental scavenging and less controlled antibiotic usage compared to organized systems. These results support the broader hypothesis in veterinary epidemiology that extensive farming systems, characterized by varied environmental exposures, tend to harbor more heterogeneous and environmentally-derived ARG profiles compared to the highly structured, therapeutically-driven resistomes of intensive indoor farms [27].

The virome of both groups was dominated by *Caudoviricetes* (bacteriophages), which are critical for horizontal gene transfer. Buffaloes exhibited greater viral diversity, with unique detection of *Alsuviricetes*, *Pokkesviricetes*, and *Herviviricetes*. Buffaloes showed a significantly richer virome, with 4895 viral contigs (227 per sample average) compared to 1750 in bovines (175 per sample average). *Caudoviricetes* has been widely reported as the most prevalent class of prophage families in the rumen, and *S. aureus* NP01 bacteriophage, also a *Caudoviricetes*, has been isolated from raw cattle milk [28]. More recently, *Caudoviricetes* prophages are being explored as a biocontrol therapy for cattle mastitis [29] as well as for preserving milk and decontaminating it from *B. cereus* [30]. The richer and more diverse viral community in buffaloes suggests a greater potential for phage-mediated transduction of genetic material, including AMR genes, aligning with emerging evidence that bacteriophages play a substantial role in AMR dissemination within complex agricultural matrices [31].

We detected clinically relevant resistance genes. *AbaQ*, found in cattle, encodes an MFS efflux pump linked to quinolone resistance [32]. ArnT, also detected, mediates lipid A modification, reducing susceptibility to colistin, a last-resort drug [33]. In buffaloes, the transcriptional regulator *LeuO* was identified; this pleiotropic factor modulates efflux systems and decreases susceptibility to multiple antibiotic classes. Furthermore, the detection of *KpnF* (an SMR efflux component) in buffalo milk highlights the potential for efflux-mediated resistance to disseminate beyond clinical contexts [34,35,36,37].

The resistome analysis reveals distinct host-associated risk profiles. Bovine samples were dominated by vancomycin resistance clusters (*vanB*/*G*/*I*). As vancomycin is a critical last-resort antibiotic for human medicine (e.g., MRSA treatment), its reservoir in cattle is of significant zoonotic concern. Conversely, buffaloes presented a resistome enriched for fosfomycin (*FosA* variants) and polymyxin (*ArnT*, *PmrF*) resistance. The high prevalence of *FosA* genes and multi-AMR contigs (e.g., *KpnE* + *KpnF*) in buffaloes suggests a complex reservoir where mobile genetic elements likely facilitate the accumulation of resistance determinants. The recovery of last-resort resistance determinants—such as those for vancomycin, colistin, and fosfomycin—in our dataset parallels alarming global trends where critical ARGs are increasingly detected in food-producing animals, often pointing to widespread environmental contamination rather than direct clinical application [38,39].

The co-occurrence of ARGs with MGEs is of particular concern. While our bioinformatic pipeline did not directly link ARGs to predicted plasmid contigs, this is a well-known limitation of short-read metagenomics. ARGs are often flanked by repetitive elements that break the assembly graph, separating resistance genes from their defining plasmid backbone markers. The detection of classically plasmid-associated genes in dairy environments, such as *blaZ* and *tet* determinants, strongly suggests that plasmid-mediated AMR is present in these microbiomes but was masked by assembly fragmentation. Furthermore, the high prevalence of integrons and the detection of triple co-occurrences (AMR + plasmid signatures + viral signatures) within the dataset support the hypothesis of complex plasmid-phage-AMR networks facilitating gene mobility in these dairy environments [35,40,41,42]. Similar correlations between MGEs and ARGs have been reported by other researchers in dairy microbiomes, underscoring the high mobility potential of these resistance networks despite methodological limitations [43].

While this study provides a comprehensive regional snapshot of the raw milk resistome in the Amazon, it is important to acknowledge the limitations of the pooling strategy employed. Pooling 250 individual samples into 32 composites was logistically necessary but introduces a dilution effect. Consequently, high-concentration ARG “hotspots” originating from specific high-risk animals or farms may have been diluted by lower-load samples, potentially masking localized peaks of AMR and causing rare resistance elements to fall below the detection threshold of shotgun metagenomics. Future studies should aim to address AMR distribution at the farm and animal levels by employing a targeted approach: initial phenotypic screening for high-resistance milk samples, followed by individual shotgun metagenomics or Whole-Genome Sequencing (WGS) of resistant isolates. This would enable a higher resolution of the genotype-phenotype correlations driving AMR persistence in these local dairy systems.

## 4. Materials and Methods

### 4.1. Study Design and Sampling

A cross-sectional study was conducted over a seven-month period (February to August 2019) to investigate raw milk samples from dairy operations supplying three key milk processing facilities in the Manaus Metropolitan Region, Amazonas state, Brazil. Sampling was performed opportunistically by collecting two 50-mL aliquots from 50-L milk containers upon arrival at processing plants. Milk shipments were delivered directly from farms without intermediate storage in bulk tanks. Samples were transported under refrigeration (4 °C) and stored frozen (−20 °C) until laboratory processing.

### 4.2. Characterization of Sampled Herds

The sampled dairy farms operated under extensive to semi-intensive production systems. Extensive herds typically comprised fewer than 100 lactating cows with manual milking, whereas semi-intensive herds generally exceeded 100 lactating cows and employed mechanical milking. Farmers in extensive systems generally lacked comprehensive veterinary care but maintained access to antibiotics via local agricultural supply stores. Conversely, semi-intensive systems usually utilized regular veterinary assistance. Because systematic farm-level record-keeping of veterinary treatments is practically nonexistent in this region, quantitative data on antibiotic usage could not be collected. However, qualitative observational data gathered during farm visits revealed that partially used or stockpiled antibiotic bottles (typically broad-spectrum agents) were frequently present in both systems, often used empirically by farmers to treat conditions such as mastitis and omphalitis without veterinary prescription.

### 4.3. Sample Pooling and DNA Extraction

From 250 individual milk samples (91 bovine, 159 buffalo), samples were aggregated into pools based on species and geographic origin due to logistical constraints. This resulted in 32 composite samples (10 bovine, 22 buffalo). DNA isolation followed a modified phenol-chloroform protocol adapted for milk matrices. Briefly, 50-mL milk samples were centrifuged, and equal volumes of the pellet and upper fat layer were lysed with SDS and proteinase K. Following phenol:chloroform:isoamyl alcohol purification and isopropanol precipitation, DNA was resuspended in ultrapure water. DNA concentration and purity were assessed using a Qubit 4.0 Fluorometer (Thermo Fisher Scientific (Waltham, MA, USA) and a NanoDrop spectrophotometer (Thermo Fisher Scientific, Waltham, MA, USA) prior to library preparation.

### 4.4. Metagenomic Sequencing and Bioinformatics

Shotgun metagenomic sequencing (SMS) was performed at the FIOCRUZ Next-Generation Sequencing Platform (Rio de Janeiro, Brazil) using the Nextera DNA Flex Library Prep Kit (Illumina, Inc., San Diego, CA, USA) and HiSeq Rapid SBS Kit v2 (200 cycles). Raw reads were quality-checked (FASTQC v0.12.0) and trimmed (Trim Galore v0.6.5). Metagenomic assembly was performed using SPAdes (v4.2.0, metaSPAdes mode). ARGs were identified using the Resistance Gene Identifier (RGI v6.0.4) with the Comprehensive Antibiotic Resistance Database (CARD v4.0.1). Plasmids were identified using PlasmidHunter v1.0.0 and geNomad v1.2.0. Viral sequences were identified using geNomad. Integrons were detected using IntegronFinder v2.

### 4.5. Statistical Analysis

Descriptive statistics were employed to characterize genomic data, including arithmetic mean and median for contig size distributions, and range (minimum-maximum) for dispersion. Box plots were used to visualize interquartile ranges and identify outliers in contig distributions. Comparative analyses used frequency counts for categorical classifications (multi-AMR gene combinations, co-occurrence patterns, rare events) and percentage rates for no-result contigs.

## 5. Conclusions

This study confirms that raw cow and buffalo milk from the Amazonas state harbor diverse antimicrobial resistance genes (ARGs) and mobile genetic elements (MGEs), identifying these dairy matrices as potential hotspots for horizontal gene transfer. Notably, buffalo milk exhibited a higher abundance and diversity of resistance determinants, including complex multi-AMR gene cassettes, likely facilitated by a richer microbial community and abundant mobilome activity. The persistence of these elements is primarily driven by regional farm-level practices—particularly extensive to semi-intensive systems with limited veterinary oversight and easy access to antibiotics, which create selective pressure favoring ARGs. Furthermore, the distinct resistome profiles observed, vancomycin markers in cattle versus fosfomycin/polymyxin markers in buffaloes, suggest divergent, species-specific selection pressures. Given the cultural habit of consuming unpasteurized dairy products in the Brazilian Amazon, these findings highlight a direct route for human AMR exposure. This poses a significant public health concern within a One Health framework, underscoring the urgent need for improved antimicrobial stewardship, stricter sanitary enforcement, and continuous, species-specific surveillance in the regional dairy chain.

## Data Availability

The raw shotgun metagenomic sequencing data generated and analyzed in this study have been deposited in the NCBI Sequence Read Archive (SRA) and are publicly available under the BioProject accession number PRJNA1432505. Supplementary Data has been deposited at Harvard Dataverse https://doi.org/10.7910/DVN/2GV0TY.

## Supplementary Materials

The following supporting information can be downloaded at: https://doi.org/10.7910/DVN/2GV0TY (accessed on 27 March 2026), File S1: AMR results from bovines; File S2: AMR results from buffaloes; File S3: All analysis results from bovines sample; File S4: All analysis results from buffaloes samples.
